# An Enhanced Artificial Bee Colony Algorithm with Solution Acceptance Rule and Probabilistic Multisearch

**DOI:** 10.1155/2016/8085953

**Published:** 2015-12-24

**Authors:** Alkın Yurtkuran, Erdal Emel

**Affiliations:** Department of Industrial Engineering, Uludag University, Görükle Campus, 16059 Bursa, Turkey

## Abstract

The artificial bee colony (ABC) algorithm is a popular swarm based technique, which is inspired from the intelligent foraging behavior of honeybee swarms. This paper proposes a new variant of ABC algorithm, namely, enhanced ABC with solution acceptance rule and probabilistic multisearch (ABC-SA) to address global optimization problems. A new solution acceptance rule is proposed where, instead of greedy selection between old solution and new candidate solution, worse candidate solutions have a probability to be accepted. Additionally, the acceptance probability of worse candidates is nonlinearly decreased throughout the search process adaptively. Moreover, in order to improve the performance of the ABC and balance the intensification and diversification, a probabilistic multisearch strategy is presented. Three different search equations with distinctive characters are employed using predetermined search probabilities. By implementing a new solution acceptance rule and a probabilistic multisearch approach, the intensification and diversification performance of the ABC algorithm is improved. The proposed algorithm has been tested on well-known benchmark functions of varying dimensions by comparing against novel ABC variants, as well as several recent state-of-the-art algorithms. Computational results show that the proposed ABC-SA outperforms other ABC variants and is superior to state-of-the-art algorithms proposed in the literature.

## 1. Introduction

Optimization techniques play an important role in the field of science and engineering. Over the last five decades, numerous algorithms have been developed to solve complex optimization algorithms. Since more and more present-day problems turn out to be nonlinear, multimodal, discontinuous, or dynamic in nature, derivative-free, nonexact solution methods attract ever-increasing attention. Evolutionary biology or swarm behaviors inspired most of these methods. There have been several classes of algorithms proposed in this evolutionary or swarm intelligence framework including genetic algorithms [1, 2], memetic algorithms [3], differential evolution (DE) [4], ant colony optimization (ACO) [5], particle swarm optimization (PSO) [6], artificial bee colony algorithm (ABC) [7], cuckoo search [8], and firefly algorithm [9].

The ABC is a biologically inspired population-based metaheuristic algorithm that mimics the foraging behavior of honeybee swarms [7]. Due to its simplicity and ease of application, the ABC has been widely used to solve both continuous and discrete optimization problems since its introduction [10]. It has been shown that ABC tends to suffer poor intensification performance on complex problems [11–13]. To improve the intensification performance of ABC, many researchers have focused on the search rules as they control the tradeoff between diversification and intensification. Diversification means the ability of an algorithm to search for unvisited points in the search region, whereas intensification is the process of refining those points within the neighborhood of previously visited locations to improve solution quality. Various new search strategies, mostly inspired from PSO and DE, have been proposed in the literature. Zhu and Kwong [14] proposed a global best guided ABC, which utilizes the global best individual's information within the search equation similar to PSO. Gao et al. [15] introduced another variant of global best ABC. Inspired by DE, Gao and Liu [13] introduced a modified version of the ABC in which ABC/Best/1 and ABC/Rand/1 were employed as local search equations. Kang et al. [16] described the Rosenbrock ABC, which combines Rosenbrock's rotational method with the original ABC. To improve diversification, Alatas [11] employed chaotic maps for initialization and chaotic searches within a search strategy. Akay and Karaboga [17] introduced a modified version of the ABC in which frequency of perturbation is controlled adaptively and the ratio of variance operator was introduced. Liao et al. [18] proposed a detailed experimental analysis and comparison of an ABC variant with different search equations. Gao et al. [19] introduced two new search equations for onlooker and employed bee phases and a new robust comparison technique for candidate solutions. ABC. Qiu et al. [20] were inspired from DE/current-to-best/1 strategy in DE algorithm and proposed a modified ABC. Banitalebi et al. [21] proposed an enhanced compact ABC, which did not store the actual population of candidate solution; instead their approach employed probabilistic representation. Wang et al. [22] presented multistrategy ABC, in which a pool of different search strategies was constructed and various search strategies were used during the search process. Gao et al. [23] introduced a bare bones ABC with parameter adaptation and fitness-based neighborhood to improve the intensification performance of standard ABC. Ma et al. [24] reduced the redundant search moves and maintained the diversity of the swarm by introducing hybrid ABC with life cycle and social learning. Furthermore, ABC has been successfully applied to solve various types of optimization problems, such as production scheduling [25, 26], vehicle routing [27], location-allocation problem [28], image segmentation [29], wireless sensor network routing [30], leaf-constrained minimum spanning tree problem [31], clustering problem [32], fuel management optimization [33], and many others [34–36]. Readers can refer to Karaboga et al. [10] for an extensive literature review of the ABC and its applications.

This study presents an enhanced ABC with solution acceptance rule and probabilistic multisearch (ABC-SA) in order to solve global optimization problems efficiently. In ABC-SA, three search mechanisms with different diversification and intensification characteristics are employed. Moreover, search mechanism selection probabilities *p*_*s*1_, *p*_*s*2_, and *p*_*s*3_ are introduced to control the balance between diversification and intensification. In our proposed approach, a search mechanism is established using the selection probabilities to generate a new neighbor solution from the current one. Additionally, a solution acceptance rule is implemented, in which not only better solutions but also worse solutions may be accepted by using a probability function. A nonlinearly decreasing acceptance probability function is employed, thus allowing worse solutions to be more likely accepted in the early phases of the search. Therefore, ABC-SA algorithm explores the search space more widespread, especially in the early phases of the search process. By using solution acceptance rule and implementing different search mechanisms of contrasting nature, ABC-SA balances the trade-off between diversification and intensification efficiently. The proposed approach is tested on six different benchmark functions with varying dimensions and compared to novel ABC, PSO, and DE variants. Computational results reveal that ABC-SA outperforms competitor algorithms in terms of solution quality.

The main contributions of the proposed study are as follows:

- Three different search mechanisms are employed with varying diversification and intensification abilities. Probabilistic multisearch with predetermined probability values are employed to determine the search mechanism to be used to generate candidate solutions. Therefore, ABC-SA explores and exploits the search space efficiently.
- Instead of a greedy selection, a new candidate solution acceptance rule is integrated, where a worse solution may have a chance to be accepted as new solution. By the help of this new acceptance rule, ABC-SA achieves better diversification performance, specifically in the early phases of the search.

The remainder of this paper is structured as follows: Section 2 presents the traditional ABC; Section 3 introduces the proposed framework; the instances, parameter settings, and computational results are presented in Section 4 and finally Section 5 concludes the paper.

## 2. Artificial Bee Colony Algorithm

The ABC has inspired from the organizational nature and foraging behavior of honeybee swarms. In the ABC algorithm, the bee colony comprises three kinds of bees: employed bees, onlooker bees, and scout bees. Each bee has a specialized task in the colony to maximize the nectar amount that is stored in the hive. In ABC, each food source is placed in the *D*-dimensional search space and represents a potential solution to the optimization problem. The amount of nectar in the food source is assumed to be the fitness value of a food source. Generally, the number of employed and onlooker bees is the same and equal to the number of food sources.

Each employed bee belongs to a food source and is responsible for mining the corresponding food source. Then, employed bees pass the nectar information to onlooker bees in the “dance area.” Onlooker bees wait in the hive and select a food source to mine based on the information coming from the employed bees. Here, more beneficial food sources will have higher selection probabilities to be selected by onlooker bees. In ABC, in order to decide if a food source is abandoned or not, trial counters and a predetermined limit parameter are used. If a solution represented by a food source does not improve during a number of trials (limit), the food source is abandoned. When the food source is abandoned, the corresponding employed bee will become a scout bee and randomly generate a new food source and replace it with the abandoned one.

The ABC algorithm consists of four main steps: initialization, employed bee phase, onlooker bee phase, and scout bee phase. After the initialization step, the other three main steps of the algorithm are carried out repeatedly in a loop until the termination condition is met. The main steps of the ABC algorithm are as follows.

**Step 1 (initialization).**

In the initialization step, the ABC generates a randomly distributed population of* SN* solutions (food sources), where* SN* also denotes the number of employed or onlooker bees. Let *x*_*i*_ = {*x*_*i*,1_, *x*_*i*,2_,…, *x*_*i*,*D*_} represent the *i*th food source, where *D* is the problem size. Each food source is generated within the limited range of *j*th index by

(1) $$\begin{array}{c} x_{i,j}=x_{j}^{min}+\varphi_{i,j}\left(\;x_{j}^{max}-x_{j}^{min}\;\right), \end{array}$$

where *i* = 1, 2,…, *SN*, *j* = 1, 2,…, *D*,   *φ*_*i*,*j*_ is a uniformly distributed random real number in [0,1], and *x*_*j*_^min^ and *x*_*j*_^max^ are the lower and upper bounds for the dimension *j*, respectively. Moreover, a trial counter for each food source is initialized.

**Step 2 (employed bee phase).**

In the employed bee phase, each employed bee visits a food source and generates a neighboring food source in the vicinity of the selected food source. Employed bees search a new solution, *v*_*i*_, by performing a local search around each food source *i* = 1, 2,…, *SN* as follows:

(2) $$\begin{array}{c} v_{i,j}=x_{i,j}+\phi \left(\;x_{i,j}-x_{r1,j}\;\right), \end{array}$$

where *j* is a randomly selected index *j* ∈ {1, 2,…, *D*} and *r*1 ∈ {1,2,…, *SN*}  is a randomly chosen food source that is not equal to *i*; that is, (*r*1 ≠ *i*). *ϕ* is a random number within the range [−1, 1] generated specifically for each *i* and *j* combination. A greedy selection is applied between *x*_*i*_ and *v*_*i*_ by selecting the better one.

**Step 3 (onlooker bee phase).**

Unlike the employed bees, onlooker bees select a food source depending on the probability value *p*, which is determined by nectar amount associated with that food source. The value of *p*_*i*_ is calculated for *i*th food source as follows:

(3) pi=fiti∑j=1SNfitj,

(4) fiti=11+fi,fi≥01+absfi,fi<0,

where fit_*i*_ is the fitness value of solution *i* and calculated as in (4) for minimization problems. Different fitness functions are employed for maximization problems. By using this type of roulette wheel based probabilistic selection, better food sources will more likely be visited by onlooker bees. Therefore, onlooker bees try to find new candidate food sources around good solutions. Once the onlooker bee chooses the food source, it generates a new solution using (2). Similar to the employed bee phase, a greedy selection is carried out between *x*_*i*_ and *v*_*i*_.

**Step 4 (scout bee phase).**

A trial counter is associated with each food source, which depicts the number of tries that the food source cannot be improved. If a food source cannot be improved for a predetermined number of tries (limit) during the onlooker and employed bee phases, then the employed bee associated with that food source becomes a scout bee. Then, the scout bee finds a new food source using (1). By implementing the scout bee phase, the ABC algorithm easily escapes from minimums and improves its diversification performance.

**Figure 1:**
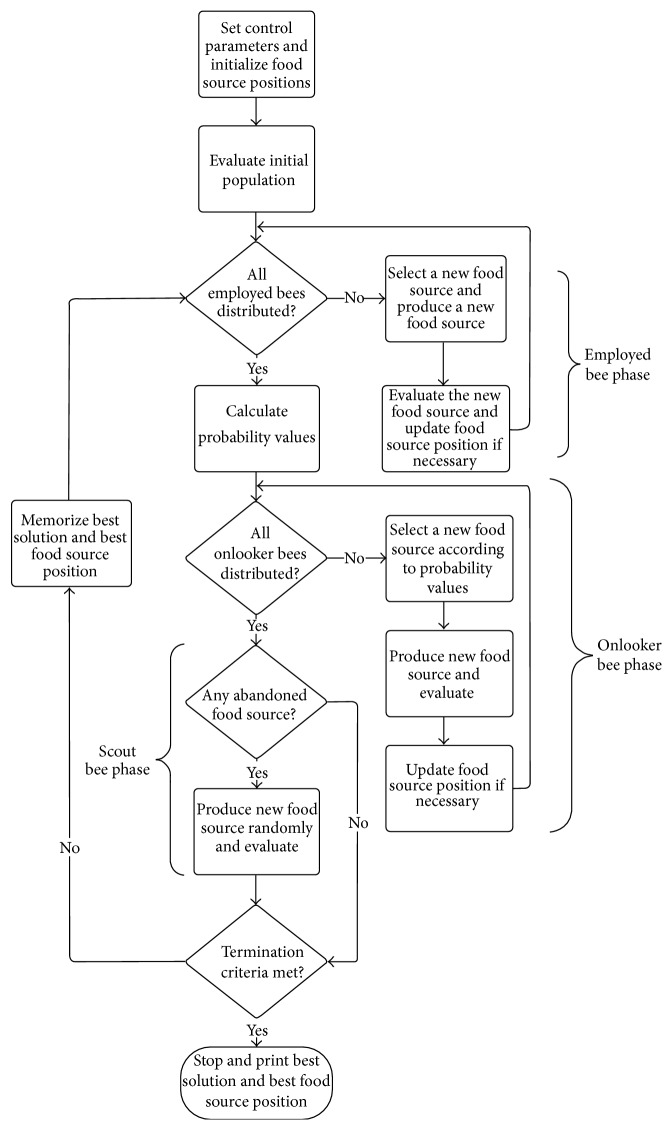
Flowchart of ABC.

It should be noted that, in the employed bee phase, a local search is applied to each food source, whereas in the onlooker bee phase better food sources will more likely be updated. Therefore, in ABC algorithm, the employed bee phase is responsible for diversification whereas the onlooker bee phase is responsible of intensification. The flow chart of the ABC is given in Figure 1.

## 3. Proposed Framework

In this section, the proposed algorithm is described in detail. First, a solution acceptance rule is presented. Second, a novel probabilistic multisearch mechanism is proposed. Finally, the complete ABC-SA mechanism is given.

### 3.1. Solution Acceptance Rule

**Figure 2:**
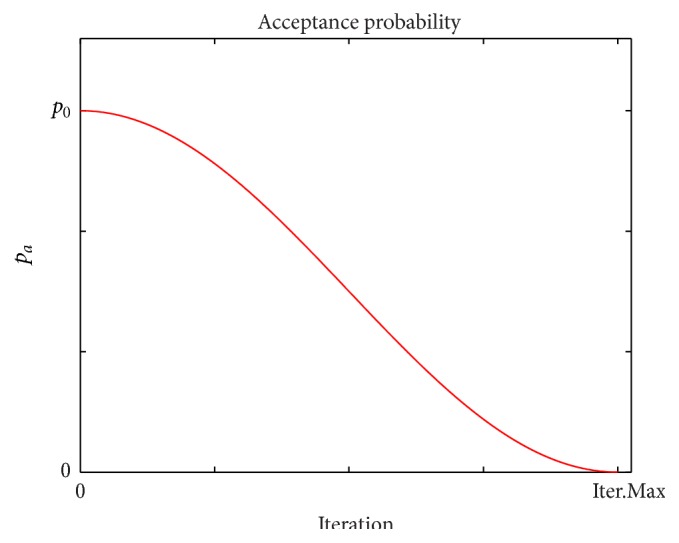
Acceptance probability curve.

In order to strengthen the diversification ability of ABC-SA mechanism, a solution acceptance rule is proposed. Instead of greedy selection in both employed and onlooker bee phases, an acceptance probability is given to worse solutions. The main idea behind this acceptance probability is not to restrict the search moves to only better solutions. By accepting a worse solution, the procedure may escape from a local optimum and explore the search space effectively. In ABC-SA algorithm, if a worse solution is generated, it is accepted if the following condition holds:

(5) r<pa

(6) pa=po1+cos⁡iter/Max.iterπ2,

where *r* is a random real number within [0,1], *p*_*a*_ is the acceptance probability, *p*_*o*_ denotes the initial probability, and iter and Max.iter represent the current iteration number and the maximum iteration number, respectively. According to (6), the acceptance probability *p*_*a*_ is nonlinearly decreased from *p*_0_ to zero during the search process. As can be seen from (6), *p*_*a*_ = 0 when iter = Max.iter and the range of *p*_*a*_ is [0, *p*_0_]. A typical *p*_*a*_ graph is given in Figure 2 and Algorithm 1 presents the implementation of the solution acceptance rule. At this point, it is important to note that the trial counter is incremented, whether a worse candidate solution is accepted or not.

### 3.2. Probabilistic Multisearch Strategy

In standard ABC, a candidate solution is generated using the information of the parent food source with the guidance of the term *ϕ*_*i*,*j*_(*x*_*i*,*j*_ − *x*_*r*1,*j*_) in (3). However, there is no guarantee that a better individual influences the candidate solution; therefore, it is possible to have a poor convergence speed and intensification performance. In fact, studying search equations is a trending topic to improve the ABC's performance. Recently, numerous search equations have been proposed, such as [13–16, 19, 20, 37, 38]. It is well known that the balance between diversification and intensification is the most critical part of any metaheuristic algorithm.

In ABC-SA approach, instead of employing a single search mechanism throughout the search process, a probabilistic multisearch mechanism with three different search rules is used. A probabilistic selection is employed using predefined probability parameters to select the search rule within both employed and onlooker bee phases. The three search rules which were proposed by [7, 13, 14], respectively, are presented as follows:

(7) vi,j=xi,j+ϕxi,j−xr1,j,

(8) vi,j=xi,j+ϕxi,j−xr1,j+ψxgbest,j−xi,j,

(9) vi,j=xlbest,j+ϕxi,j−xr1,j,

where *i* is a food source, *j* is a randomly selected index for all *i* = 1, 2,…, *SN*, and *j* ∈ {1, 2,…, *D*}, respectively. *r*1 is a randomly chosen food source where *r*1 ≠ *i*. *gbest* stands for the global best solution, whereas *lbest* is the best solution in the current population. *ϕ* represents a real random number within the range of [−1, 1] [7]. Finally, *ψ* is a real random number within the range of [0, *C*] where *C* is a predetermined number [13].

Equation (7) is the original search rule, which was discussed in previous sections. Equation (8) is presented to improve the intensification capability of ABC. Equation (8) uses the information provided by the global best solution which is similar to PSO. In (9), *lbest* guides the search with the random effect of the term *ϕ*(*x*_*i*,*j*_ − *x*_*r*1,*j*_). Equation (7) has an explorative character, whereas (9) favors intensification. On the other hand, (8) explores the search space using the second term and exploits effectively by the third term. Therefore, (8) balances diversification and intensification performance. In summary, the proposed ABC-SA uses three different search rules to achieve a trade-off between diversification and intensification. In ABC-SA, search probabilities *p*_*s*1_, *p*_*s*2_, and *p*_*s*3_ are introduced such that ∑_*k*_ *p*_*sk*_ = 1 and *k* = 1,2, 3 to select a search rule to be used in the employed and the onlooker bee phases. A roulette wheel method is employed with three cumulative ranges 0 ≤ *p*_*s*1_ ≤ *P*_*s*1_, 0 ≤ *p*_*s*2_ < *P*_*s*2_ − *P*_*s*1_, and 0 ≤ *p*_*s*3_ ≤ *P*_*s*3_ − *P*_*s*2_ assigned to (7), (8), and (9), respectively, where *P*_*s*3_ = 1.  Algorithm 2 shows the mechanism of probabilistic multisearch.

### 3.3. Proposed Approach

Algorithm 3 summarizes the ABC-SA framework. The novel parts of the ABC-SA mechanism are the probabilistic multisearch (Lines 9 and 19) and the solution acceptance rule (Lines 10 and 20) sections.

## 4. Computational Results

### 4.1. Test Instances

In literature, many test functions with different characters were used to test algorithms [11, 13, 15, 17, 19–21, 34, 37–40]. Unimodal functions have one local minimum as the global optimum. These functions are generally used to test the intensification ability of algorithms. Multimodal functions have one or more local optimums which may be the global optimum. Therefore, diversification behavior of algorithms is analyzed on multimodal instances. Separable functions can be written as sum of *n* functions with one variable, whereas nonseparable functions can not be reformulated as subfunctions. In this study, to analyze the performance of the proposed ABC-SA algorithm, 13 scalable benchmark functions with dimensions *D* = 50,  *D* = 100, and *D* = 200 are used and listed in Table 1. They are Rosenbrock, Ackley, Rastrigin, Weierstrass, Schwefel 2.26, Shifted Sphere, Shifted Schwefel 1.2, Shifted Rosenbrock, Shifted Rastrigin, Step, Penalized 2, and Alpine. In Table 1, function label, name, formulation, type (UN: unimodal and nonseparable, MS: multimodal and separable, and MN: multimodal and nonseparable), range, and optimal values (*f*(*x* ^*∗*^)) are given.

### 4.2. Parameters Settings

Parameter settings may have a great influence on the computational results. The ABC-SA mechanism has seven control parameters such as maximum iteration number (Max.iter), limit, population size (*SN*), *p*_*s*1_, *p*_*s*2_, *ψ*, and *p*_0_. Maximum iteration number is the termination condition, and *p*_0_ is the initial acceptance probability. First, Max.iter is set to 4,000, limit = 0.2 × *D* × *SN*, where *D* is the dimension of the problem [21], *SN* is taken to be 40 for 50*D* and 100*D* problems and 50 for 200*D* problems [40], and *ψ* is set to be a random real number within (0, 1.5) [14]. Then, preliminary experiments were conducted with appropriate combinations of the following parameter values to determine the best settings:

 
*p*_0_ = 0.25, 0.20, 0.15, 0.10, and 0.05,
 
*p*_*s*1_ = 0.2, 0.4, and 0.6,
 
*p*_*s*2_ = 0.2, 0.4, and 0.6,
 
*p*_*s*3_ = 0.2, 0.4, and 0.6.

From the results of the pilot studies, *p*_0_ = 0.10, *p*_*s*1_ = 0.20, *p*_*s*2_ = 0.60, and *p*_*s*3_ = 0.20 settings achieved the best results. Therefore, these parameter settings are used for further experiments.

### 4.3. Comparison with ABC Variants

In this section, aforementioned ABC-SA is implemented and evaluated by benchmarking with other well-known ABC variants including the original ABC [7], GABC [14], and IABC [13] on problems F1–F13.

The parameters of test algorithms are set to their original values given in their corresponding papers, except for the maximum number of function evaluations, population size, and limit, which are set to the same values for all ABC variants. ABC-SA, ABC, and GABC implement random initialization mechanisms whereas IABC employs a chaotic initialization as described in [13]. All algorithms have been simulated in MATLAB environment and executed on the same computer with Intel Xeon CPU (2.67 GHz) and 16 GB of memory.

The computational results are presented in Table 2 for 50*D* problems, Table 3 for 100*D* problems, and Table 4 for 200*D* problems. In Tables 2–4, results are given in terms of mean and standard deviation of the objective values due to the repetitive runs for the global best solutions. All algorithms were run 30 times with random seeds and the stopping criteria set to 4,000 iteration, which means that 320,000 functions evaluations for 50*D* and 100*D* problems and 400,000 function evaluations for 200*D* problems approximately. For a precise and pairwise comparison, statistical significances of the differences between the means of two algorithms are analyzed using *t*-tests where significance level is set to 0.05. In Tables 2–4, “+” in the columns next to competing algorithms shows that ABC-SA outperforms the competitor algorithm, “=” indicates that the difference between the ABC-SA and the compared algorithm is not statistically significant, and “−” depicts that the competitor algorithm is better than ABC-SA at a level of 0.05 significance.

Tables 2–4 show that, according to pairwise *t* tests, ABC-SA obtains statistically better results on 25, 28, and 28 cases out of 39 comparisons for each of the 50*D*, 100*D*, and 200*D* problem types, respectively. Specifically, ABC-SA is inferior to IABC on F6 and F9 with 200*D* and GABC on F12 with 100*D*. There is no significant difference on the results that are obtained by ABC and ABC-SA on F1 (50*D* and 100*D*), F4 (100*D* and 200*D*), F7 (all dimensions), F8 200*D*, F10 50*D*, and F11 50*D*. Moreover, on F1 50*D*, F6 50*D*, F4 (100*D* and 200*D*), F7 (50*D* and 100*D*), F8 200*D*, F10 (50*D* and 100*D*), F11 50*D*, and F13 100*D*, GABC and ABC-SA perform statistically similar. Further, ABC-SA and IABC perform equally well, namely, on F1 50*D*, F6 (50*D* and 100*D*), F7 (all dimensions), F8 200*D*, F10 (all dimensions), F11 50*D*, and F13 100*D*. Standard deviation of the results also indicates that ABC-SA has a stable performance. According to the results of Tables 2–4, one can safely conclude that ABC-SA significantly surpasses ABC, GABC, and IABC on 50*D*, 100*D*, and 200*D* problems.

**Figure 3:**
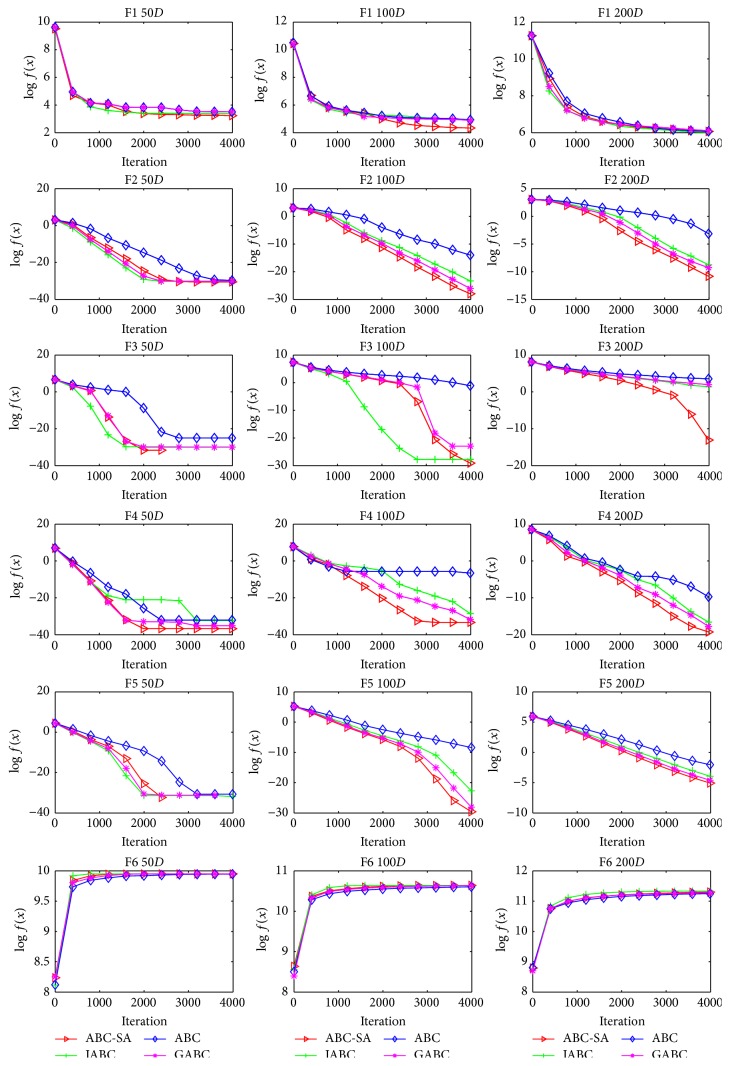
Convergence curves for ABC-SA and ABC variants.

To vividly describe the effectiveness of ABC-SA framework, the convergence curves of some benchmark problems are given in Figure 3. According to the figure, ABC-SA shows better convergence behavior on the majority of test cases when compared to ABC, GABC, and IABC.

**Figure 4:**
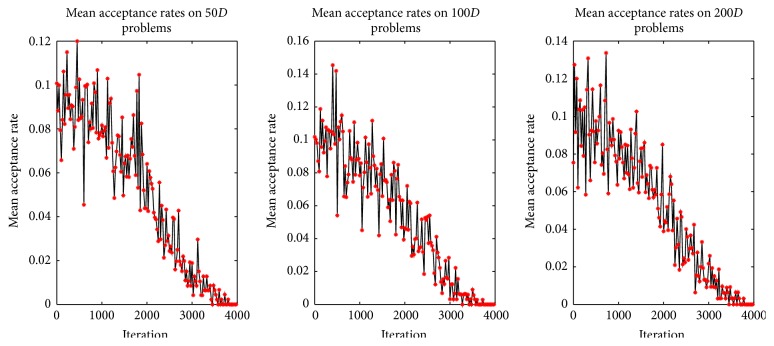
Mean acceptance rates.

Furthermore, mean acceptance rate curves for solution acceptance rule in ABC-SA framework are given in Figure 4 and the acceptance rate is determined as follows:

(10) acceptance  rate=number  of  accepted  worse  solutionstotal  number  of  worse  solutions.

The curves in Figure 4 clearly coincide with the acceptance probability curve given in Figure 2. Figure 4 also shows the nonlinear decreasing of acceptance rate throughout the search process.

### 4.4. Comparison with PSO and DE Variants

The performance of ABC-SA is also tested against novel and powerful variants of DE and PSO. The competitor algorithms are self-adapting DE (jDE) [41], adaptive DE with optional external archive (JADE) [42], self-adaptive DE (SaDE) [43], comprehensive learning PSO (CLPSO) [44], self-organizing hierarchical PSO with time-varying acceleration coefficients (HPSO-TVAC) [45], and fully informed particle swarm (FIPS) [46]. The results of these algorithms are taken directly from corresponding studies. The experimental results for 30*D* problems are shown in Tables 5 and 6 for DE and PSO variants, respectively. Some of the benchmarks problems are not included in the comparisons, since results on these problems were not reported in competitor studies. The previous parameter setting for ABC-SA is used, but this time maximum function evaluation number (Max.FE) is employed as the stopping criteria. Since the results of competitor algorithms are taken directly from corresponding studies, statistical significance tests could not be applied. Therefore, in this part of the analysis, mean and standard deviations of results are compared directly. In Tables 5 and 6, the winner algorithms are indicated in bold character according to the mean results of 30 independent runs. As can be seen from Tables 5 and 6, ABC-SA outperforms other algorithms on all cases, except in the case of F2, F4, and F12. SaDE performs better than the ABC-SA on F4 and HPSO-TVAC outperforms ABC-SA on only F2 and F12. ABC achieves better results on the majority of the instances in terms of robustness according to the standard deviations of the results. These results also indicate the effectiveness of ABC-SA when compared to other novel swarm based and evolutionary algorithms.

## 5. Conclusion and Future Work

This paper presents a modified ABC algorithm, namely, the ABC-SA, enhanced with a solution acceptance rule and a probabilistic multisearch strategy. In ABC-SA, instead of a greedy selection, a new acceptance rule is presented, where a worse candidate solution has a probability to be accepted. Furthermore, to balance the diversification and intensification tendency of the algorithm, a probabilistic multisearch mechanism is employed. In the probabilistic multisearch, a search rule is selected among three alternatives according to their predetermined probabilities. The proposed algorithm is very effective as compared to other novel ABC variants and state-of-the-art algorithms. Several experimental studies are conducted and results show that ABC-SA outperforms all other competitor algorithms on the majority of the test cases. Future research will be along the line of implementing the ABC-SA to solve complex engineering problems.

## Figures and Tables

**Algorithm 1:**
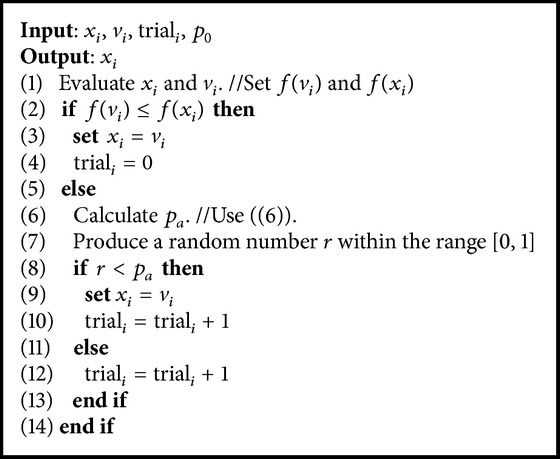
Solution acceptance rule.

**Algorithm 2:**
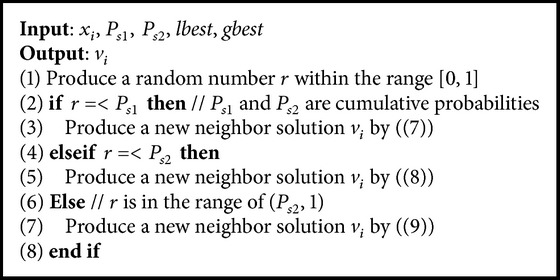
Probabilistic multisearch.

**Algorithm 3:**
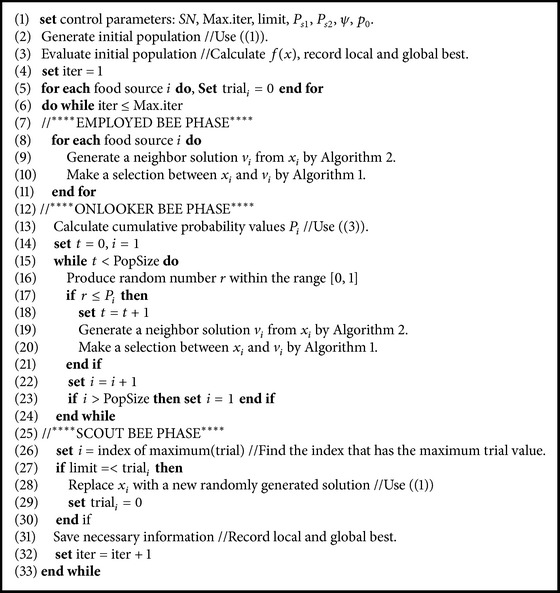
ABC-SA framework.

**Table 1:** Test functions used in experiments.

Label	Name	Formulation	Type	Range	*f*(*x* ^*∗*^)
F1	Rosenbrock	$f_{1}\left(\vec{X}\right)=\sum_{i=1}^{D-1}\left[{100\left(x_{i+1}-x_{i}^{2}\right)}^{2}+{\left(x_{i}-1\right)}^{2}\right]$	UN	[−2.048, 2.048]^*D*^	0
F2	Ackley	$f_{2}\left(\vec{X}\right)=-20\exp\left(-0.2\sqrt{\frac{1}{D}}\sum_{i=1}^{D}x_{i}^{2}\right)-\exp\left(\frac{1}{D}\sum_{i=1}^{D}\cos\left(2\pi x_{i}\right)\right)+20+e$	MS	[−32.768, 32.768]^*D*^	0
F3	Rastrigin	$f_{3}\left(\vec{X}\right)=\sum_{i=1}^{D}\left[x_{i}^{2}-10\cos\left(2\pi x_{i}\right)+10\right]$	MS	[−5.12, 5.12]^*D*^	0
F4	Griewank	$f_{4}\left(\vec{X}\right)=\frac{1}{4000}\sum_{i=1}^{D}x_{i}^{2}-\prod_{i=1}^{D}\cos\left(\frac{x_{i}}{\sqrt{i}}\right)+1$	MN	[−600, 600]^*D*^	0
F5	Weierstrass	$f_{5}\left(\vec{X}\right)=\sum_{i=1}^{D}\left(\sum_{k=0}^{k_{\max}}\left[a^{k}cos\left(2\pi b^{k}\left(x_{i}+0.5\right)\right)\right]\right)-D\sum_{k=0}^{k_{\max}}\left[a^{k}cos\left(2\pi b^{k}0.5\right)\right],\;a=0.5,\;\;b=3,\;\;k_{\max}=20$	MS	[−0.5, 0.5]^*D*^	0
F6	Schwefel 2.26	$f_{6}\left(\vec{X}\right)=418.9829\times D-\sum_{i=1}^{D}-x_{i}\sin\left(\sqrt{\left|x_{i}\right|}\right)$	MS	[−500, 500]^*D*^	−418.98 × *D*
F7	Shifted Sphere	$f_{7}\left(\vec{X}\right)=\sum_{i=1}^{D}z_{i}^{2}-f_{\text{bias}},\;z=x-o,\;\;f_{\text{bias}}=-450$	US	[−100, 100]^*D*^	*f*_bias_
F8	Shifted Schwefel 1.2	$f_{8}\left(\vec{X}\right)=\sum_{i=1}^{D}\left(\sum_{j=1}^{i}z_{j}\right)+f_{\text{bias}},\;z=x-o,\;\;f_{\text{bias}}=-450$	UN	[−100, 100]^*D*^	*f*_bias_
F9	Shifted Rosenbrock	$f_{9}\left(\vec{X}\right)=\sum_{i=1}^{D-1}\left(100{\left(z_{i}^{2}-z_{i+1}\right)}^{2}+{\left(z_{i}-1\right)}^{2}\right)+f_{\text{bias}},\;z=x-o+1,\;\;f_{\text{bias}}=390$	MN	[−100, 100]^*D*^	*f*_bias_
F10	Shifted Rastrigin	$f_{10}\left(\vec{X}\right)=\sum_{i=1}^{D}\left[z_{i}^{2}-10\cos\left(2\pi z_{i}\right)+10\right]+f_{\text{bias}},\;z=x-o,\;\;f_{\text{bias}}=-330$	MS	[−5, 5]^*D*^	*f*_bias_
F11	Step	$f_{11}\left(\vec{X}\right)=\sum_{i=1}^{D}\left({\left\lfloor x_{i}+0.5\right\rfloor }^{2}\right)$	US	[−100, 100]^*D*^	0
F12	Penalized 2	$f_{12}\left(\vec{X}\right)=\frac{1}{10}\left\{\sin^{2}\left(\pi x_{1}\right)+\sum_{i=1}^{D-1}{\left(x_{i}-1\right)}^{2}\left[1+\sin^{2}\left(3\pi x_{i+1}\right)\right]+{\left(x_{n}-1\right)}^{2}\right\}$ [1 + sin⁡^2^(2*πx*_*i*+1_)] + ∑_*i*=1_^*D*^ *u*(*x*_*i*_, 5,100,4)	MN	[−50, 50]^*D*^	0
F13	Alpine	$f_{13}\left(\vec{X}\right)=\sum_{i=1}^{D}\left|x_{i}\cdot \sin\left(x_{i}\right)+0.1\cdot x_{i}\right|$	MS	[−10, 10]^*D*^	0

**Table 2:** Comparisons of ABC-SA and ABC variants on 50*D* problems.

Func.	ABC-SA		ABC			GABC			IABC		
	Mean	Std. Dev.	Mean	Std. Dev.	Sign	Mean	Std. Dev.	Sign	Mean	Std. Dev.	Sign
F1	3.10*E* + 01	1.18*E* + 01	3.84*E* + 01	1.07*E* + 01	=	3.32*E* + 01	6.71*E* + 00	=	3.25*E* + 01	1.75*E* + 01	=
F2	5.30*E* − 14	4.10*E* − 15	1.17*E* − 13	1.62*E* − 14	+	7.96*E* − 14	1.18*E* − 15	+	7.44*E* − 14	9.63*E* − 15	+
F3	0.00*E* + 00	0.00*E* + 00	2.02*E* − 11	7.11*E* − 12	+	8.53*E* − 14	4.16*E* − 14	+	8.53*E* − 14	3.58*E* − 14	+
F4	1.11*E* − 16	2.17*E* − 16	4.78*E* − 12	2.61*E* − 13	+	6.11*E* − 16	3.62*E* − 16	+	1.26*E* − 13	6.80*E* − 14	+
F5	0.00*E* + 00	0.00*E* + 00	3.84*E* − 14	1.90*E* − 14	+	8.53*E* − 15	9.94*E* − 15	+	1.71*E* − 14	8.99*E* − 15	+
F6	−2.09*E* + 04	2.51*E* − 15	−2.09*E* + 04	6.09*E* + 00	+	−2.09*E* + 04	4.05*E* − 11	=	−2.09*E* + 04	5.57*E* − 14	=
F7	−4.50*E* + 02	0.00*E* + 00	−4.50*E* + 02	6.36*E* − 14	=	−4.50*E* + 02	2.84*E* − 14	=	−4.50*E* + 02	4.92*E* − 14	=
F8	1.92*E* + 04	5.19*E* + 03	3.22*E* + 04	1.79*E* + 03	+	3.61*E* + 04	5.89*E* + 03	+	2.92*E* + 04	5.21*E* + 03	+
F9	3.98*E* + 02	3.11*E* + 00	5.03*E* + 02	3.96*E* + 00	+	4.13*E* + 02	2.45*E* + 01	+	4.23*E* + 02	2.48*E* + 01	+
F10	−3.30*E* + 02	0.00*E* + 00	−3.30*E* + 02	3.14*E* − 14	=	−3.30*E* + 02	0.00*E* + 00	=	−3.30*E* + 02	1.02*E* − 14	=
F11	0.00*E* + 00	0.00*E* + 00	0.00*E* + 00	0.00*E* + 00	=	0.00*E* + 00	0.00*E* + 00	=	0.00*E* + 00	0.00*E* + 00	=
F12	4.69*E* − 15	1.90*E* − 16	5.90*E* − 14	9.82*E* − 15	+	1.93*E* − 14	4.77*E* − 15	+	8.77*E* − 15	7.43*E* − 15	+
F13	3.69*E* − 24	9.34*E* − 26	2.95*E* − 23	3.01*E* − 23	+	7.71*E* − 24	3.90*E* − 25	+	6.53*E* − 24	1.66*E* − 25	+

**Table 3:** Comparisons of ABC-SA and ABC variants on 100*D* problems.

Func.	ABC-SA		ABC			GABC			IABC		
	Mean	Std. Dev.	Mean	Std. Dev.	Sign	Mean	Std. Dev.	Sign	Mean	Std. Dev.	Sign
F1	7.65*E* + 01	4.76*E* + 01	1.26*E* + 02	3.55*E* + 01	+	1.35*E* + 02	2.95*E* + 01	+	1.34*E* + 02	2.27*E* + 01	+
F2	6.16*E* − 13	9.63*E* − 14	6.97*E* − 07	3.25*E* − 07	+	4.56*E* − 12	1.18*E* − 12	+	7.06*E* − 11	1.31*E* − 14	+
F3	2.27*E* − 13	5.41*E* − 13	3.97*E* − 01	5.81*E* − 01	+	2.37*E* − 11	5.97*E* − 11	+	1.21*E* − 12	6.69*E* − 13	+
F4	1.58*E* − 14	2.64*E* − 14	1.30*E* − 04	7.13*E* − 04	=	7.73*E* − 10	3.90*E* − 09	=	3.54*E* − 12	9.27*E* − 12	+
F5	1.48*E* − 13	4.40*E* − 14	1.64*E* − 04	4.98*E* − 05	+	7.62*E* − 13	3.24*E* − 13	+	1.20*E* − 10	1.25*E* − 11	+
F6	−4.19*E* + 04	6.84*E* + 01	−4.06*E* + 04	2.61*E* + 02	+	−4.16*E* + 04	1.53*E* + 02	+	−4.19*E* + 04	6.24*E* + 01	=
F7	−4.50*E* + 02	1.90*E* − 14	−4.50*E* + 02	1.81*E* − 13	=	−4.50*E* + 02	8.53*E* − 14	=	−4.50*E* + 02	4.90*E* − 14	=
F8	8.85*E* + 04	1.00*E* + 03	1.61*E* + 05	2.43*E* + 04	+	1.60*E* + 05	1.15*E* + 04	+	1.95*E* + 05	7.92*E* + 03	+
F9	3.89*E* + 02	9.55*E* + 00	3.95*E* + 02	1.04*E* + 01	+	4.14*E* + 02	4.12*E* + 01	+	4.10*E* + 02	1.76*E* + 01	+
F10	−3.30*E* + 02	0.00*E* + 00	−3.22*E* + 02	9.41*E* − 04	+	−3.30*E* + 02	7.20*E* − 07	=	−3.30*E* + 02	5.68*E* − 14	=
F11	1.17*E* + 01	5.24*E* + 00	4.32*E* + 01	6.43*E* + 00	+	9.10*E* + 01	6.92*E* + 00	+	2.08*E* + 01	9.52*E* + 00	+
F12	9.12*E* − 13	1.04*E* − 15	5.54*E* − 11	7.27*E* − 13	+	8.71*E* − 13	6.90*E* − 14	−	1.15*E* − 12	3.91*E* − 13	+
F13	1.52*E* − 20	5.48*E* − 19	8.14*E* − 19	9.17*E* − 20	+	7.03*E* − 20	4.45*E* − 19	=	8.44*E* − 20	1.06*E* − 19	=

**Table 4:** Comparisons of ABC-SA and ABC variants on 200*D* problems.

Func.	ABC-SA		ABC			GABC			IABC		
	Mean	Std. Dev.	Mean	Std. Dev.	Sign	Mean	Std. Dev.	Sign	Mean	Std. Dev.	Sign
F1	4.06*E* + 02	3.34*E* + 01	4.32*E* + 02	4.21*E* + 01	=	4.47*E* + 02	5.89*E* + 01	+	4.58*E* + 02	6.77*E* + 01	+
F2	1.85*E* − 05	3.30*E* − 05	4.44*E* − 02	2.13*E* − 02	+	6.68*E* − 05	1.65*E* − 05	+	1.47*E* − 04	3.69*E* − 05	+
F3	8.27*E* − 06	2.53*E* − 05	3.17*E* + 01	5.89*E* + 00	+	6.51*E* + 00	1.74*E* + 00	+	4.02*E* + 00	1.03*E* + 00	+
F4	6.05*E* − 10	2.26*E* − 09	5.75*E* − 04	2.58*E* − 03	=	1.79*E* − 08	5.29*E* − 08	=	6.05*E* − 08	4.68*E* − 08	+
F5	6.42*E* − 03	4.93*E* − 04	1.24*E* − 01	1.35*E* − 02	+	9.76*E* − 03	1.21*E* − 03	+	1.83*E* − 02	1.40*E* − 03	+
F6	−8.19*E* + 04	1.00*E* + 02	−7.63*E* + 04	7.61*E* + 02	+	−7.89*E* + 04	4.20*E* + 02	+	−8.37*E* + 04	3.68*E* + 02	−
F7	8.56*E* + 05	1.81*E* + 04	8.60*E* + 05	1.46*E* + 04	=	8.73*E* + 05	2.49*E* + 04	+	8.62*E* + 05	1.75*E* + 04	=
F8	2.89*E* + 06	9.10*E* + 04	2.91*E* + 06	2.07*E* + 05	=	2.93*E* + 06	3.13*E* + 05	=	2.98*E* + 06	3.90*E* + 05	=
F9	7.63*E* + 11	4.37*E* + 08	8.02*E* + 11	5.05*E* + 09	+	7.87*E* + 11	4.72*E* + 08	+	7.61*E* + 11	4.93*E* + 08	−
F10	3.74*E* + 03	4.19*E* + 01	3.78*E* + 03	5.94*E* + 01	+	3.76*E* + 03	5.55*E* + 01	+	3.76*E* + 03	4.60*E* + 01	=
F11	7.34*E* + 02	4.40*E* + 02	8.82*E* + 03	9.22*E* + 02	+	5.39*E* + 03	9.03*E* + 02	+	1.04*E* + 03	6.01*E* + 02	+
F12	3.79*E* − 11	4.90*E* − 13	1.91*E* − 09	9.41*E* − 14	+	4.88*E* − 11	2.25*E* − 12	+	6.22*E* − 11	3.89*E* − 12	+
F13	9.55*E* − 18	1.04*E* − 18	5.02*E* − 15	1.55*E* − 15	+	4.44*E* − 16	5.03*E* − 17	+	1.92*E* − 17	7.33*E* − 18	+

**Table 5:** The comparisons of ABC-SA and DE variants on 30*D* problems.

Func.	Max.FE	ABC-SA		jDE		JADE		SaDE	
		Mean	Std. Dev.	Mean	Std. Dev.	Mean	Std. Dev.	Mean	Std. Dev.
F1	300,000	1.19**E** − 01	7.91**E** − 02	1.30*E* + 01	1.40*E* + 01	3.20*E* − 01	1.10*E* + 00	2.10*E* + 01	7.70*E* + 01
F2	50,000	3.01**E** − 09	7.44**E** − 10	2.37*E* − 04	7.10*E* − 05	3.35*E* − 09	2.84*E* − 09	3.81*E* − 06	8.26*E* − 07
F3	100,000	2.77**E** − 09	6.09**E** − 10	2.37*E* − 04	7.10*E* − 05	3.35*E* − 09	2.84*E* − 09	3.81*E* − 06	8.26*E* − 07
F4	50,000	7.23*E* − 09	4.11*E* − 09	7.29*E* − 06	1.05*E* − 05	1.57*E* − 08	1.09*E* − 07	2.52**E** − 09	1.24**E** − 09
F6	100,000	8.63**E** − 11	4.94**E** − 11	1.70*E* − 10	2.62*E* − 10	2.62*E* − 04	3.59*E* − 04	1.13*E* − 08	1.08*E* − 08
F11	10,000	4.41**E** + 00	3.90**E** + 00	6.13*E* + 02	1.72*E* + 02	5.62*E* + 00	1.87*E* + 00	5.07*E* + 01	1.34*E* + 01
F12	50,000	1.41**E** − 10	7.23**E** − 11	1.80*E* − 05	1.42*E* − 05	1.87*E* − 10	1.09*E* − 09	1.93*E* − 09	1.53*E* − 09
F13	300,000	4.99**E** − 11	2.49**E** − 10	6.08*E* − 10	8.36*E* − 10	2.78*E* − 05	8.43*E* − 06	2.94*E* − 06	3.47*E* − 06

**Table 6:** The comparisons of ABC-SA and PSO variants on 30*D* problems.

Func.	Max.FE	ABC-SA		FIPS		HPSO-TVAC		CLPSO	
		Mean	Std. Dev.	Mean	Std. Dev.	Mean	Std. Dev.	Mean	Std. Dev.
F1	200,000	7.61**E** + 00	2.18**E** − 01	2.51*E* + 01	5.10*E* − 01	2.39*E* + 01	2.65*E* + 01	1.13*E* + 01	9.85*E* + 00
F2	200,000	4.01*E* − 13	1.05*E* − 13	2.33*E* − 07	7.19*E* − 08	7.29**E** − 14	3.00**E** − 14	3.66*E* − 07	7.57*E* − 08
F3	200,000	6.91**E** − 10	9.44**E** − 11	6.51*E* + 01	1.33*E* + 01	9.43*E* + 00	3.48*E* + 00	9.05*E* − 05	1.25*E* − 04
F4	200,000	8.89**E** − 12	6.56**E** − 13	9.01*E* − 12	1.84*E* − 11	9.75*E* − 03	8.33*E* − 03	9.02*E* − 09	8.57*E* − 09
F6	200,000	1.79**E** − 12	8.89**E** − 11	9.93*E* + 02	5.09*E* + 02	1.59*E* + 03	3.26*E* + 02	3.82*E* − 04	1.28*E* − 05
F11	200,000	0.00*E* + 00	0.00*E* + 00	0.00*E* + 00	0.00*E* + 00	0.00*E* + 00	0.00*E* + 00	0.00*E* + 00	0.00*E* + 00
F12	200,000	5.03*E* − 23	1.44*E* − 24	2.70*E* − 14	1.57*E* − 14	2.79**E** − 28	2.18**E** − 28	1.25*E* − 12	9.45*E* − 12
